# Machine Learning Techniques for Undertaking Roundabouts in Autonomous Driving

**DOI:** 10.3390/s19102386

**Published:** 2019-05-24

**Authors:** Laura García Cuenca, Javier Sanchez-Soriano, Enrique Puertas, Javier Fernandez Andrés, Nourdine Aliane

**Affiliations:** Science, Computing and Technology, Universidad Europa de Madrid, Calle Tajo s/n, Villaviciosa de Odón, 28670 Madrid, Spain; javier.sanchez@universidadeuropea.es (J.S.-S.); enrique.puertas@universidadeuropea.es (E.P.); javier.fernandez@universidadeuropea.es (J.F.A.); nourdine.aliane@universidadeuropea.es (N.A.)

**Keywords:** machine learning, autonomous driving, sensor fusion, data mining, roundabouts, deep learning, support vector machines, linear regression

## Abstract

This article presents a machine learning-based technique to build a predictive model and generate rules of action to allow autonomous vehicles to perform roundabout maneuvers. The approach consists of building a predictive model of vehicle speeds and steering angles based on collected data related to driver–vehicle interactions and other aggregated data intrinsic to the traffic environment, such as roundabout geometry and the number of lanes obtained from Open-Street-Maps and offline video processing. The study systematically generates rules of action regarding the vehicle speed and steering angle required for autonomous vehicles to achieve complete roundabout maneuvers. Supervised learning algorithms like the support vector machine, linear regression, and deep learning are used to form the predictive models.

## 1. Introduction

At the beginning of 2015, the UK considered the possibility of autonomous vehicles circulating in a shared traffic and controlled traffic environment for the first time. By the end of the same year, the Spanish traffic agency (DGT) had launched an administrative instruction (15/V-113) [1] allowing the tests for research on self-driving vehicles to be carried out on roads. This is also allowed in many other countries in the word, where several international organizations, such as SAE (Society of Automotive Engineers) [2] and USDOT (United States Department of Transportation) [3], have defined frameworks for autonomous vehicles. Among these frameworks, automated driving is divided into five levels according to the level of human intervention, where the fifth level corresponds to a completely autonomous vehicle without a driver. The four previous levels require more or less driver intervention.

An ideal autonomous vehicle is one driven by a system behaving like a human driver would [4], where the driving model transforms information perceived from different driving situations into actions on the vehicle’s actuators, such as its steering wheel or pedals [5]. It is necessary to understand driving and driver behaviors under different driving circumstances, so that the correct actions to be taken can be predicted during different driving circumstances [6] as well as to design an ADAS (Advanced Driver Assistance Systems) for delivering adaptable alarms [7] and to allow the vehicle to undertake corrective actions during automated driving [8]. Autonomous driving is complex, since the cars involved are subject to factors such as sharing the road with other vehicles and the distance and/or speed of the obstacles present in the traffic environment, to cite a few examples [9]. Autonomous driving starts by understanding naturalistic driving, where drivers are faced with real and complex scenarios [10]. One of these challenging traffic scenarios is driving in roundabouts [11]. 

In the last 20 years, Spanish urban and interurban roads roundabouts have been replacing intersections progressively with the idea of improving traffic flow [12]. However, roundabouts have become one of the most conflicting traffic scenarios [13,14]. In fact, studies [15,16] carried out by the DGT (Spanish Traffic Agency) between 2012 and 2016 concluded that accidents in roundabouts increased by almost 34%, and according to Reference [17], the number of fatal accidents in roundabouts doubled over the same period. Furthermore, studies such as References [18,19] concluded that drivers have not learned the driving rules in roundabouts, which is why roundabouts become a constant source of traffic jams and accidents.

Driving in roundabouts has been a subject of research during recent years, and understanding the maneuvers within roundabouts is a complicated task, since various factors, such as driver–car interactions and environment perception, are involved [20]. For example, roundabout management and design are addressed in works such as References [21,22] and how to estimate traffic flow is covered in References [23,24]. Onboard computer-based vision systems are also used as a solution in [25,26] to analyze driving scenes and to extract information about traffic in roundabouts. They have also been used for the creation of reference points to simultaneously detect and classify traffic signals to train the networks at the point of reference [27], to detect high precision 3D objects during autonomous driving using a multitier sensory fusion model using the LIDAR point cloud [28], and to generate an algorithm that combines 3D point clouds and 2D images to detect and recognize traffic signals based on BoVP (bag-of visual-phrases) and deep hierarchical models [29]. Further, their use as an approach to fuse information from multiple sources while addressing the uncertainty present in the perception process itself is discussed in Reference [30]. Other relevant issues in self-driving vehicles are related to character and text recognition from images processing [31], or generation of motion models of the scene (background and foreground) from video frames of a moving camera [32]. In Reference [33] a combination of the two previous concepts is presented: the separation of text from background in images and the separation of moving objects from a background undergoing global camera motion in videos. In Reference [34] there is a similar approach focused on path planning, where the solution consists in extracting static obstacles from depth maps computed out of multiple consecutive images.

Another approach for understanding driving in roundabouts is via exploring data mining and artificial intelligence techniques. More specifically, Reference [35] presents an approach for driving classification based on support vector machines (SVM) and hidden Markov models (HMM) machine learning algorithms trained with naturalistic driving data. In Reference [36] an attempt was made to model roundabout conduction through data obtained from external cameras installed to capture the circulation activity and the entry behavior to these roundabouts, focusing on vehicle speed and traffic in the roundabouts. In Reference [37] roundabout safety is analyzed in circumstances where autonomous vehicles are mixed with conventional vehicles, and models are based on speed and traffic. Reference [24] presents the use of SVM and addresses predicting vehicle intention in terms of staying inside or exiting the roundabout, where the study employs GPS, steering angle sensor, and odometer for data acquisition, and uses steering angle as a feature attribute. A similar research is presented in Reference [38], investigating the impact of roundabout layout on driving behavior, where data used are from a driving simulator. In contrast to the commented previous work, such as Reference [36] based data taken from external cameras, or [37] estimating only vehicle speed according to traffic, or [24] aimed at predicting driver intention to stay or leave roundabout, or [38] the impact of roundabout layout, the present paper uses real-driving datasets built from driving along several roundabouts with different diameters, number of lanes, and during different traffic and weather conditions. Furthermore, the study is aimed at estimating two variables: vehicle speed and steering angle.

The present article presents a technique for generating rules of action for autonomous vehicles to undertake maneuvers in roundabouts. These rules of actions are the result of modeling the driver’s behavior while taking several factors into account. Some of them are specific to driver–vehicle interactions, such as moving the steering wheel, braking, or accelerating. Others are intrinsic to the traffic environment, such as roundabout geometry or the number of lanes. This paper focuses, firstly, on the deployment of the necessary instrumentation for data acquisition, and secondly, on the processing of the acquired data for drivers by modeling using computational techniques based on different machine learning algorithms, namely, the support vector machine, linear regression, and deep learning algorithms.

The remainder of the article is organized as follows: Section 2 describes the driving maneuvers in roundabouts according to Spanish rules and the subsequent problems that autonomous vehicles might face. Section 3 presents the developed on-board experimental setup used for data acquisition. Section 4 explains the datasets used to model driver training. Section 5 discuss the computational techniques used to construct rules of action that autonomous vehicles must follow to perform roundabout maneuvers. The results and validation rules are also presented. Finally, conclusions as well as the future work of the present research are given in Section 6.

## 2. Roundabout Driving Rules in Spain

**Figure 1:**
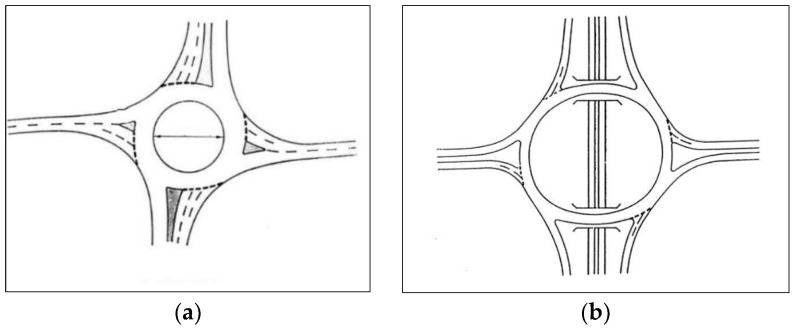
Roundabouts topologies, (**a**) standard roundabout (**b**) roundabout at different levels with two bridges.

**Figure 2:**
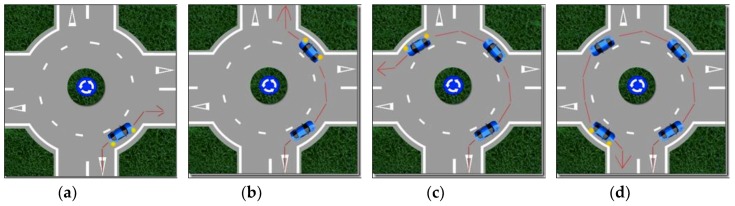
Correct movement of a vehicle in a roundabout: (**a**) first exit, (**b**) second exit, (**c**) third exit, (**d**) fourth exit.

According to the traffic and road safety rules in Spain [39], roundabout denomination designates a special type of intersection characterized by several converging road sections that communicate through a ring in which a rotating circulation is established around a central island (see Figure 1). The basic circulation in a roundabout is achieved by leaving the central island to the left-hand-side of the driver. Vehicles inside the road have preference over the incorporating vehicles, despite arriving at the right side. This implies that the general rule of priority to the right-hand-side does not fully apply in roundabouts. Trajectories within a roundabout converge and diverge, but do not cross. Figure 2 shows the correct movement of a vehicle within roundabouts graphically.

The principal problems that an autonomous car might face when undertaking roundabouts are three points: the choice of the roundabout entrance lane, applying priority rules inside a roundabout, and exiting the roundabout. This paper focuses on understanding these issues and proposes rules of action for autonomous vehicles and how they should undertake roundabout maneuvers.

## 3. Hardware Setup for Data Collection

**Figure 3:**
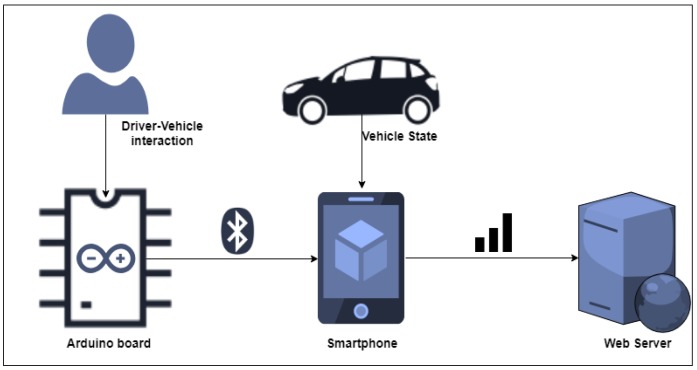
Architecture for data collection based on three components: Arduino, Smartphone, and server.

In order to collect the driving data in roundabout maneuvers, data from several controls of a vehicle, such as pedals, blinkers, steering wheel spin, and other buttons, were captured and connected to a single connector accessible from the boot. These sensors were connected to a data acquisition module based on an Arduino board that was in charge of reading the state of the sensors in the loop, composing a data frame, and sending it by Bluetooth to a smartphone application (APP). This APP was developed to measure the information provided by the sensors of the smartphone such as GPS (Global Positioning System), accelerometers, a gyroscope, and a camera. This raw information was pre-processed by generating a data frame ordered by the timestamp which, along with the videos of the different routes, was temporarily stored in the phone until it was sent to the web server. This server centralized data from different drivers and processed it to its final storage form in a database. The architecture of the system is summarized in Figure 3, and the different elements of this architecture are detailed in the following paragraphs.

### 3.1. Vehicle Sensors

**Figure 4:**
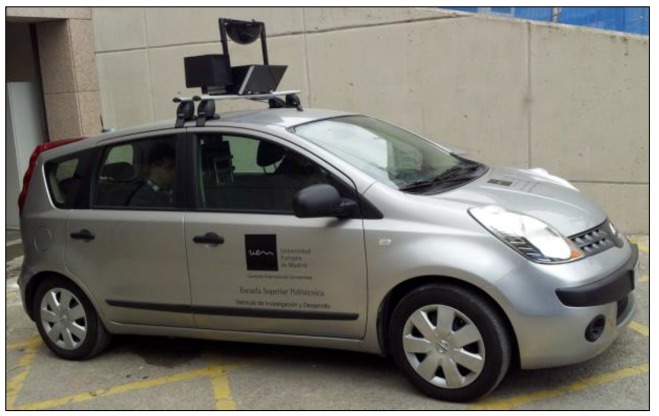
Test vehicle: Nissan Note 1.4 Visia.

The vehicle used in this work was a utilitarian tourism 2007 Nissan Note (see Figure 4). Its main controls, such as pedals and blinkers, were captured by contact sensors. Also, the wheel rotation was obtained by means of a relative 7-bit encoder located on the steering wheel axis that was able to encode up to three turns of the flywheel. All this instrumentation was fed with current from the car’s own wiring. The information collected was the following:

- Accelerator—measured in two states: the accelerator pedal is pressed or not.
- Brake—measured in two states: the brake pedal is pressed or not.
- Clutch—measured in two states: the clutch pedal is pressed or not.
- Direction rotation—measured in decimal values of the direction spin. In the vehicle used, the angles of the wheels with respect to the longitudinal axis of the vehicle were –40 to 40. An encoder with 128 states (EAW-Absolute contacting encoder), which offers a resolution of 0.625 with respect to the orientation of the guiding wheels, was used.
- Left blinker—measured in two states: the left blinker is active or not.
- Right-flashing—measured in two states: the right blinker is active or not.
- Emergency lights—measured in two states: emergency lights are active or not.
- Auxiliary pushbuttons 1 and 2—measures in two states: the button is active or not. They can be used for many purposes and are located on the dashboard in a very accessible position for the driver and co-driver.

The information was collected at a value of 12 V, from the digital output provided by the encoder (7-bit) to the signals corresponding to the current connections that feed the blinkers, emergency lights, the exits of the pedal or pushbutton limit switches. All these signals were encapsulated in a hose-type cable with a multi-pin connector for further cleaning installation.

### 3.2. Arduino Board

**Figure 5:**
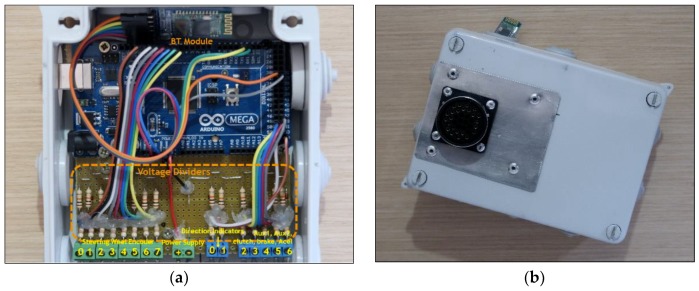
(**a**) Arduino Mega board and its associated electronics for data collection; (**b**) box with connection hose connector.

The Arduino board is used to capture the driver’s interactions with the vehicle, including signals related to the vehicle’s braking, clutch, and acceleration pedals, the right and left indicators of the vehicle, and the steering wheel rotation. The sampling frequency was set to 2 Hz, which is appropriate for the collection of data associated with car navigation and driver interactions with the vehicle. The Arduino board was fed directly to the vehicle’s battery through its DC connectors. The board had a built-in voltage regulator, a guaranteed input voltage range of 7 to 12 V, and no additionally required electronic components. The different sensors were connected to voltage dividers, which reduced the 12 V in the outputs of the sensors to 5 V, at which the board worked. Finally, the board was connected to the Smartphone via Bluetooth. Figure 5 shows the Arduino board, its protection box, and the view of the board connector.

### 3.3. Smartphone

A smartphone was used to collect other information for the experiment. It was attached to the windscreen by means of a suitable gripping device for this purpose and with the camera pointing forward. This phone ran an application designed for this purpose. The information collected was as follows:

- GPS location—the position of the vehicle was collected by using an integrated GPS receiver on the smartphone. The parameters collected were latitude, longitude (geographic coordinates format, in degrees and fractions), and altitude (above sea level).
- Accelerometer—the data from the vehicle’s accelerometer were collected, which allowed the accelerations as well as their inclination to be determined. In particular, the measures collected were the usual ones from this type of component: acceleration along the X, Y, and Z axes without any bias compensation and acceleration along the X, Y, and Z axes with estimated bias compensation. In both cases the units were m/s^2^.
- Video—video signals were collected from the vehicle’s dashboard.

**Figure 6:**
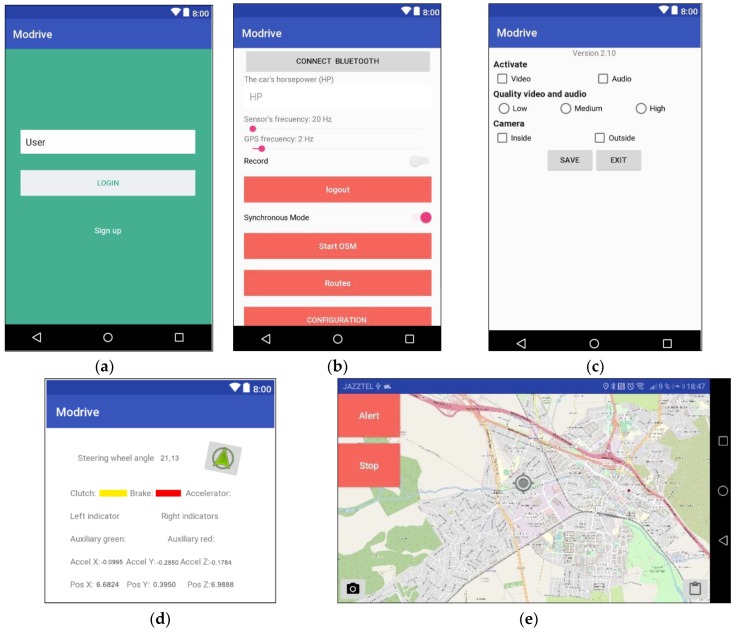
Data collection APP screens: (**a**) user ID; (**b**) basic route data and options menu; (**c**) video and audio settings; (**d**) view of vehicle status in the application. Specifically, this includes the information regarding data captured by the Arduino board (steering wheel rotation, pedal status, intermittent, and auxiliary buttons) as well as data from the Smartphone’s accelerometer. (**e**) View of data capture in route.

The application allowed us to view all of the registered parameters and the routes in a map. Finally, the collected data can be sent to the web server for storage and future processing. Figure 6 shows some screenshots related to the different functionalities of the APP.

### 3.4. Web Server

**Figure 7:**
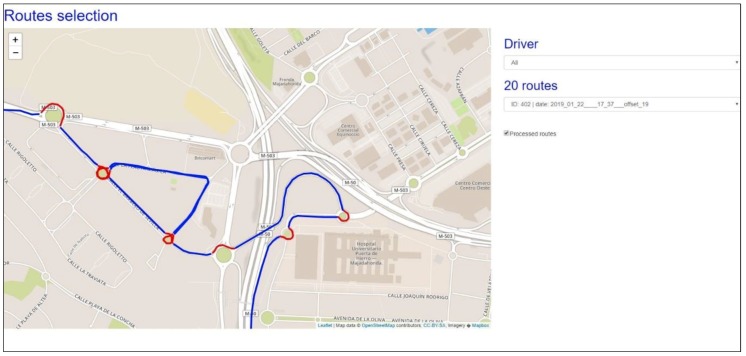
View of the vehicle’s status in the application. Specifically, the information regarding data captured by the Arduino board (steering wheel rotation, pedal status, intermittent, and auxiliary buttons) as well as data from the Smartphone’s accelerometer are shown.

The web server was used as a repository for data collection and has four important features; it acts as (1) a database for driving data, (2) a route viewer, (3) an API (application programming interface) for communication with the mobile application and the route viewer, and (4) a processing module for aggregating generated data. The server is based on XAMPP (Apache distribution containing MariaDB, PHP, and Perl) architecture and was installed on a Windows server in the author’s institution. The database is hosted on a server and contains tables with user information, routes, and information collected during driving. The route viewer has a main section in which a map is displayed, as well as some filters allow the user to search by driver or by route. The API for communication with the application and the Web Viewer is based on a PHP framework and allows database queries, the visualization of information, and its storage when receiving information from the mobile APP. Figure 7 shows an example of capturing the retrieval of information related to a route, showing the route and the roundabouts (marked in red).

## 4. Dataset Preparation

This section describes the process of data acquisition and the process of generating the aggregated data as well as segmenting it to prepare the final dataset used for the model training process.

### 4.1. Data Acquisition Process

Several drivers participated in the data acquisition process by driving on several roads in the metropolitan area of Madrid during a period of three months, including routes with roundabouts with different diameters and with single and multiple lanes. A total of 230 journeys were performed at different times of the day as well as under different weather conditions. All the drivers used the same vehicle equipped with a Smartphone running the APP, as described in the previous section. On the other hand, some aggregated data were obtained by off-line processing. The aggregated parameters were as follows:

- Roundabout diameter—calculated in meters using the Open Street Maps API.
- Number of lanes within roundabouts—calculated using the Open Street Maps API.
- Vehicle speed—calculated using two consecutive GPS locations and the lasted time.
- Rain—discrete parameter to indicate a sunny (0) or rainy (1) route, obtained by off-line video post-processing.
- Night—discrete parameter to indicate daytime (0) or nighttime (1) route, obtained by off-line video post-processing.
- Traffic—discrete parameter to indicate traffic conditions: no traffic (0), car present in front (1), on the left side (2), or on the right side (3), obtained by off-line video post-processing.
- Visibility—discrete parameter to specify the visibility conditions or the degree of clearness before entering a roundabout: no visibility (0), low visibility (1), good visibility (2), or outside roundabout (3).

The roundabout diameter and the number of lanes were calculated using the “Open Street Maps” API. Queries were built by drawing a square envelope around the geographical point of the route, and attributes and associated labels, such as the roundabout diameter, number of lines, or the GPS of the circle center, were then returned. The square envelope used for queries was defined by four parameters: a minimum longitude (*x1*), a minimum latitude (*y*1), a maximum longitude (*x2*), and a maximum latitude (*y2*). These parameters were defined around a GPS point (x: longitude, y: altitude) in the route using the following relations: *x1 = x − k1; y1 = y – k1; x2 = x + k1*; and *y2 = y + k1*, where *k1* is an adjustable parameter. In this paper, a value of *k1 =* 5 was used to generate a square box with 10 m sides. See Reference [40] for further explanation on the use of this API.

**Figure 8:**
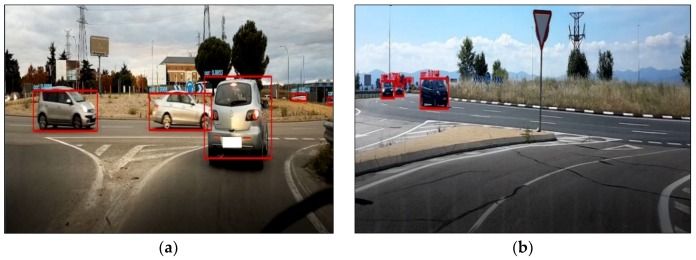
Snapshots of vehicle detection using the You Only Look Once (YOLO) framework, (**a**) Vehicles in front of and to the right, (**b**) Vehicles to the right.

Visibility and traffic parameters were obtained through video post-processing using the YOLO Framework (You Only Look Once) for object detection [41], and it was also programmed to identify lanes within roundabouts. This allowed the presence of vehicles in front of and to the right and left sides with respect to the driver’s vehicle to be detected (see Figure 8).

### 4.2. Data Segmentation

**Figure 9:**
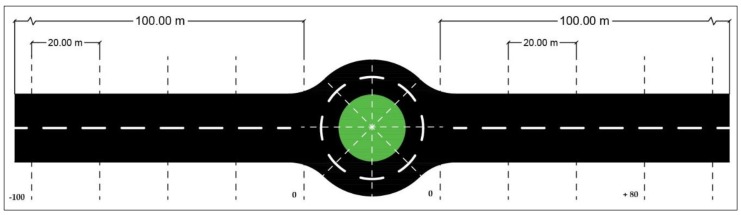
Roundabout segmentation in the before, during, and after sections.

**Figure 10:**
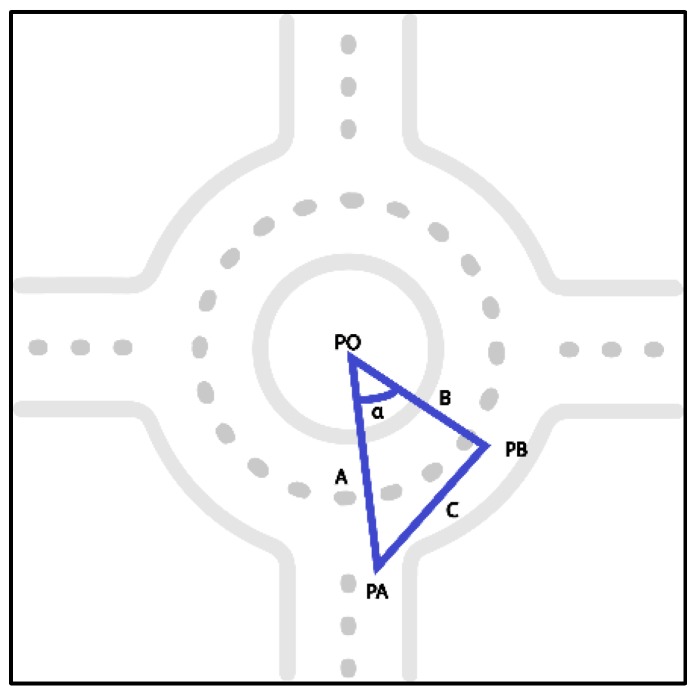
Segmentation in the 45-degree angle section within the roundabout.

In order to synthetize rules of action that should be used to complete maneuvers within roundabouts, the spacings of these roundabouts and the approaches used were divided in three sections referred to as before, during (inner), and after, as depicted in Figure 9. The section called before consisted of a segment of 100 m before the roundabout, divided in five sub-segments of 20 m. The same division was performed with the after section. All of these sub-segments were labeled with the (−) and (+) symbols depending on the distance to the roundabout. The inner section was divided into segments of 45-degree angle, taking the roundabout center and the roundabout entry point as references (see Figure 10). The calculation of sectors of 45-degree angle was based on the cosine rule. If we consider a triangle (PO, PA, PB), where PO is the roundabout center, PA is the reference point, PB is a moving point, and A, B, and C are the corresponding sides, a sector of 45 grades corresponds to distance C with the following condition: $C^{2}\;\geq \;A^{2}+B^{2}-2AB*\left(\frac{\sqrt{2}}{2}\right)$.

For the next sector, the last C point becomes a new reference and then the data records are searched to find the new C point. Finally, the whole segmentation process leads to a set of labeled segments {−100, −80, −60, −40, −20, 0, +20, +40, +60, +80, +100}, and the corresponding data are segmented accordingly.

The datasets were finally organized as a set of routes, where each route was a set of roundabouts, and each roundabout itself was segmented into sections. Data related to each section were blocks of attributes and were presented as input to different automatic learning algorithms. The list of data used for model learning is listed in Table 1.

**Figure 11:**
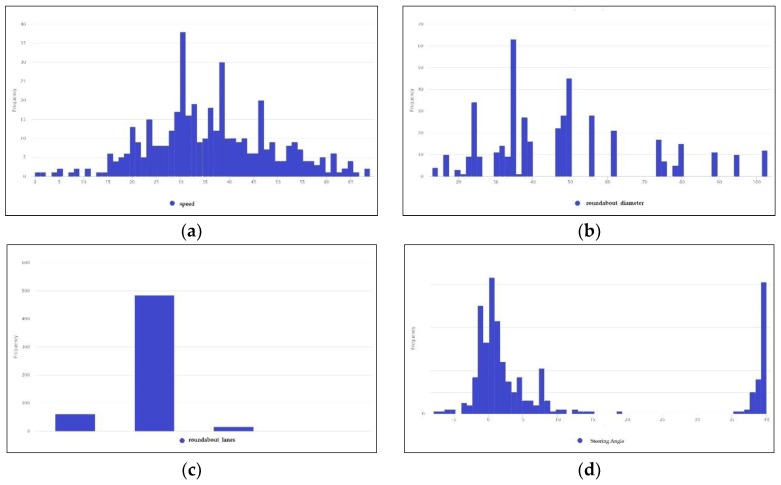
Examples of some parameter distributions: (**a**) vehicle speed, (**b**) roundabout diameters, (**c**) number of lanes within roundabouts, (**d**) steering angle.

As a final consideration, among all recorded data, 75 routes were selected, and these contained a minimum of three roundabouts each, representing a total distance of more than 20 km. Almost 80% of these routes were used during the training process. It is worth pointing out that the vehicle speeds and steering angles in the dataset corresponded to the average in each segment. The nature of data used for model training is shown in the histograms of some variables, such as the vehicle speed, roundabout diameter, number of roundabout lanes, and steering angle, as illustrated in Figure 11.

- Vehicle speed—the distribution of the vehicle speed variable, showing a speed between 0 and 69 km/h with an average speed of 36.77 km/h. The most repeated value was 31 km/h (see Figure 11a).
- Roundabout diameter—the distribution of roundabout diameters showing a small diameter of 13 m and a large diameter of 103 m, with an average value of 47.65 m. The most frequently repeated diameter was about 50 m (Figure 11b).
- Number of roundabout lanes—the distribution of the number of lanes clearly showed the number of lanes within roundabouts present in the dataset. The most used roundabouts had two lanes, as shown in Figure 11c.
- Steering angle—the distribution of steering angle showed angles ranging from –7.8 to +40, with an average of 15.7 (see Figure 11d).

## 5. Machine Learning Model

In this section, we present the algorithms used to predict the appropriate speed and steering angle for the vehicle with the current environment variables. Three machine learning techniques were used to build predictive models for rules of actions for undertaking roundabouts, namely the support vector machine (SVMs) [42], linear regression [43], and deep neural networks [44]. Deployment of training models was performed within the Rapid Miner framework [45].

### 5.1. Linear Regression

Linear regression is a technique that is used to predict numerical values like the ones that we wanted to predict in our project. It is a statistical measure that attempts to determine and weight the relationship between a dependent variable, i.e., the objective attribute, and a series of independent variables. Linear regression is used to predict numerical and continuous values like the ones that we wanted to predict in our system: speed and steering angle. Linear regression fits a model with the relationship between a scalar variable (in this case speed and steering angle) and one or more variables that store input data from vehicle data sensors by adjusting a linear equation to observed data. For our regression model, we used the avarice criterion, which measures how good a statistical model is based on measuring the data entropy.

### 5.2. Support Vector Machines

Support vector machines (SVMs) have become very popular because of the very good results obtained with these algorithms, especially when working with structured data. The SVM is a classification technique used in machine learning, but it can also be used as a regression method by maintaining all the main features that characterize the algorithm. The SVM is an algebraic method, in which maximum margin hyperplanes are built in order to attempt to separate training instances by using a kernel. Instances do not need to be linearly separable. For this work, the dot kernel was used. This kernel is the inner product of the variables and is defined as k(x,y)=x·y.

### 5.3. Deep Learning Model

The deep learning model is based on a feed-forward multilayer neural network, which is trained using stochastic gradient descent with back-propagation. The network architecture used for our model was built using two hidden layers with 50 neurons on each layer and a rectifier activation function with an adaptive learning rate that combines learning rate annealing and momentum training to avoid slow convergence.

### 5.4. Model Evaluation

A classic 80-20 stratified split of the dataset was used for validation (80% for training and 20% for testing and validation). To evaluate the training models for the three algorithms, two different metrics were used. The first one was the Root Mean Squared Error (*RMSE*), which calculates the square root of the average squared difference between the actual observation and the prediction and is given by

$$RMSE=\sqrt[{2}]{\frac{1}{N}\sum_{j=1}^{n}{\left(yi-\hat{y}i\right)}^{2}.}$$

The second one metric is the Absolute Error (*AE*), which calculates the average absolute deviation of the prediction from the actual value and is given by

$$AE=\frac{1}{n}\;\sum_{i=1}^{n}\left|y_{i}-\hat{y}i\right|.$$

After applying the different regressive algorithms, estimation of vehicle speed as well as steering angle in the different segments was performed, and this was evaluated using the metrics described previously. The results are summarized in Table 2 and Table 3.

**Figure 12:**
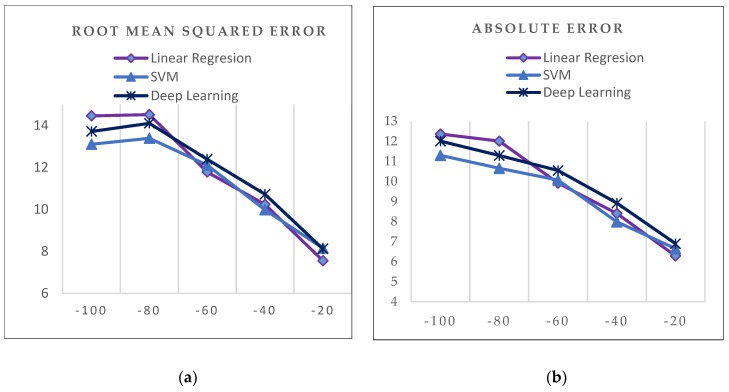

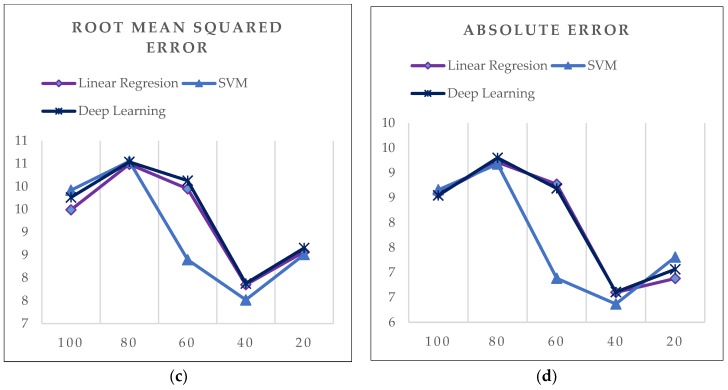
Results graph: (**a**) (RMSE) vehicle speed before segment, (**b**) (*AE*) vehicle speed before segment, (**c**) RMSE vehicle speed after segment, (**d**) *AE* vehicle speed after segment.

According to the proposed metrics, the best algorithm for predicting the vehicle speed and the steering angle was SVM, which was chosen as the reference algorithm for rule validation, as illustrated in Figure 12.

### 5.5. Generation of Rules and Validation

In the following text, some rules of action of the generated models are presented as examples. The Algorithms 1,2 are general rules of speed and steering angle estimation with driving environment variables.

**Algorithm1.** Rule for Speed Adjustment.traffic: enumerate {no-traffic, front of, left side, right side};visibility: enumerate {no-visibility, low, good, outside};rain, night: boolean;speed, steering, r_diameter, r_lanes: float;currentValues ← getSensorData()predSpeed ← predictSpeed (speedModel, currentValues)**if** (currentValues.speed > predSpeed)α ← currentValues.speed - predSpeedactivateBrake (α)
**else**
α ← predSpeed - currentValues.speedactivateAccelerator(α)
**end rule**


**Algorithm 2.** Rule for Steering Angle Adjustment.traffic: enumerate {no-traffic, front of, left side, right side};visibility: enumerate {no-visibility, low, good, outside};rain, night: boolean;speed, steering, r_diameter, r_lanes: float;currentValues ← getSensorData()predAngle ← predictSteeringAngle (steeringModel, currentValues)α ← currentValues.steering - predictSteeringAngleactivateSteeringSystem (α)
**end rule**


The cross-validation method [46] was used to validate the rules obtained in the previous section. This technique was used for model validation and to determine how the results of the statistical analysis could be generalized to independent datasets. This procedure involved repeating the calculation of the mean obtained from the evaluation obtained in different partitions of the dataset. Table 4 and Table 5 show the validation obtained using the SVM algorithm and the cross-validation for the rules presented in the previous section.

### 5.6. Discussion and Results

First of all, the work is aimed at estimating two variables to undertake roundabouts: namely, vehicle speed three different stages: when approaching, being inside, and when exiting roundabouts, and steering angle inside roundabout. The study consists mainly to obtain estimates of these two variables from naturalistic datasets built from driving along several roundabouts with different diameters, number of lanes, and during different traffic and weather conditions. As far as regressive algorithms are concerned, and according to the proposed metrics for model validation, SVM is by far the best algorithm to estimate vehicle speed and steering wheel. To improve rules of action to undertake roundabouts and model prediction, several correlation studies could be undertaken of, for example, the use of additional behavioral variables, such as acceleration, weather conditions, pedestrians, and number of lanes. Another way to improve the predictive model would be to significantly increase the number of routes and build a larger dataset with more observations. Another important aspect to be taken into account could be the parametrization of section distances, called before and after segments of roundabouts in this work, since these distances might correlate with the traffic and weather conditions. The main objective of this research was to generate rules of action for using roundabouts using different predictive models. Finally, rule validation should be carried out within a controlled environment using software simulators for autonomous driving, such as CARLA (Virtual environment to train autonomous cars) [47], for which initial work has already been undertaken.

## 6. Conclusions

In this paper, a machine-learning approach was used to build a predictive model to estimate the vehicle speed and steering angle, and to subsequently generate rules of action to be used by autonomous vehicles to perform roundabout maneuvers. Two different sets of data were used to model driver behavior: raw data acquired from an on-board instrumentation related to driver–vehicle interactions and aggregated data intrinsic to the traffic environment, such as roundabout geometry or the number of lanes obtained from Open-Street-Maps and offline video processing. The dataset used for model training and validation was organized into groups by dividing roundabouts into three sections referred to as before, during, and after segments. Three machine-learning algorithms were used for model training: namely, the support vector machine, linear regression, and deep learning algorithms. Models obtained by the support vector machine algorithm were chosen for rule validation. Validation of the model was done by means of a dataset test with a cross-validation technique. The resulting models were validated with a dataset that was not used for the model training phase. The results showed that the steering angle and vehicle speed provide important information for driving behavior prediction at specific roundabout segments.

## Figures and Tables

**Table 1:** List of parameters used for model training.

Data	Source	Data	Source
Timestamp	Smartphone	Roundabout diameter	Aggregated
Longitude	Smartphone	Number of lanes	Aggregated
Latitude	Smartphone	Vehicle speed	Aggregated
Steering angle	Vehicle	Visibility	Aggregated
Video	Smartphone	Traffic	Aggregated
		Rain	Aggregated
		Nighttime	Aggregated

**Table 2:** Comparison of the Root Mean Squared Error (*RMSE*) and Absolute Error (*AE*) metrics for vehicle speed estimation.

**Before Segment**			
**Sections**	**Linear Regression**	**Support Vector Machines (SVM)**	**Deep Learning**
–100	14,458; [12,361]	**13,106; [11,298]**	13,709; [12,007]
–80	14,517; [12,008]	**13,390; [10,655]**	14,102; [11,296]
–60	**11,777; [9,919]**	12,115; [10,062]	12,385; [10,543]
–40	10,232; [8,402]	**9,980; [7,986]**	10,720; [8,922]
–20	7,549; **[6,283]**	8,150; [6,648]	**8,117;** [6,895]
**After-Segment**			
**Sections**	**Linear Regression**	**SVM**	**Deep Learning**
+100	**9,491;** [8,588]]	9,922; [8,662]	9,762; **[8,551]**
+80	**10,490;** [9,216]	10,552; **[9,178]**	10,540; [9,304]
+60	9,952; [8,773]	**8,397; [6,883]**	10,128; [8,689]
+40	7,849; [6,599]	**7,520; [6,368]**	7,879; [6,607]
+20	8,574; **[6,879]**	**8,511;** [7,308]	8,657; [7,063]

Data are displayed in the following format: Root Mean Squared Error; [Absolute Error].

**Table 3:** Comparison of Root Mean Squared Error *(RMSE)* and Absolute Error *(AE)* metrics for vehicle speed and steering angle estimation.

Inner Segment			
	Linear Regression	Support Vector Machines (SVM)	Deep Learning
Speed	6,987; [5,135]	**6,929; [5,000]**	7,074; [5,121]
The Steering Angle	9,235; [8,216]	**8,956; [7,673]**	9,120; [7,804]

**Table 4:** Prediction of speed before and after segment—cross-validation.

***Before Segment***		
**Sections**	**Speed**	**Support Vector Machines (SVM) Prediction**
–100	53	**45,8**
–80	49	**39,7**
–60	41	**35,9**
–40	40	**32,4**
–20	25	**23,9**
***After Segment***		
**Sections**	**Speed**	**SVM prediction**
+100	46	**48,9**
+80	39	**44,1**
+60	33	**37,9**
+40	31	**36,6**
+20	23	**27,3**

**Table 5:** Prediction of speed and steering angle inner segment—cross-validation.

	Inner	Support Vector Machines (SVM) Prediction
Speed	22	**20,8**
The steering angle	0,590 (45)	**16,057 (45)**

## References

[1] Dirección General de Tráfico. http://www.dgt.es/es/.

[2] SAE—Automotive Engineers Society. https://www.sae.org.

[3] U.S. Department of Transportation. https://www.transportation.gov/.

[4] Goodrich M.A., Boer E.R. (2000). Designing Human-Centered Automation: Tradeoffs in Collision Avoidance System Design. IEEE Trans. Intell. Transp. Syst..

[5] Lefèvre S., Carvalho A., Gao Y., Tseng H.E., Borrelli F. (2015). Driver models for personalised driving assistance. Veh. Syst. Dyn. Int. J. Veh. Mech. Mobil..

[6] Chen R., Kusano K.D., Gabler H.C. (2015). Driver Behavior During Overtaking Maneuvers from the 100-Car Naturalistic Driving Study. Traffic Inj. Prev..

[7] Jamson A.H., Lai F.C.H., Carsten O.M.J. (2008). Potential benefits of an adaptive forward collision warning system. Transp. Res. Part C Emerg. Technol..

[8] Eichelberger A.H., McCartt A.T. (2014). Volvo drivers’ experiences with advanced crash avoidance and related technologies. Traffic Inj. Prev..

[9] Naranjo J.E., Sotelo M.A., Gonzalez C., García R., de Pedro T. (2007). Using Fuzzy Logic in Automated Vehicle Control. IEEE Intell. Syst..

[10] Gordon T., Srinivasan K. Modeling human lane keeping control in highway driving with validation by naturalistic data. Proceedings of the 2014 IEEE International Conference on Systems, Man, and Cybernetics (SMC).

[11] Rodrigues M., Gest G., McGordon A., Marco J. Adaptive behaviour selection for autonomous vehicle through naturalistic speed planning. Proceedings of the 2017 IEEE 20th International Conference on Intelligent Transportation Systems (ITSC).

[12] Manage S., Nakamura H., Suzuki K. (2003). Performance an of roundabouts as an alternative for intersection control in japan. J. East. Asia Soc. Transp. Stud..

[13] Abaza O.A., Hussein Z.S. Comparative analysis of multilane roundabout capacity çase study. Proceedings of the 2009 IEEE 70th Vehicular Technology Conference Fall.

[14] Akcelik R., Chung E., Besley M. (1996). Performance of Roundabouts under Heavy Demand Conditions. Road Transp. Res..

[15] Dirección General de Tráfico-Seguridad Vial. http://www.dgt.es/es/seguridad-vial/investigacion/estudios-informes/.

[16] Evolución de la Siniestralidad vial en España Fundación Mutua Madrileña. https://www.fundacionmutua.es/Estudios-de-Seguridad-Vial.html.

[17] Asociación de automovilistas. https://aeaclub.org/estudios-aea/.

[18] Yang X., Li X., Xue K. (2004). A New Traffic-Signal Control for Modern Roundabouts: Method and Application. IEEE Trans. Intell. Transp. Syst..

[19] Yang S., Jiang Y., Wang G., Deng W., Wang J. (2018). Driving Behavior Prediction at Roundabouts Based on Integrated Simulation Platform. SAE Technical Paper.

[20] Guo C., Meguro J., Kojima Y., Naito T. (2015). A Multimodal ADAS System for Unmarked Urban Scenarios Based on Road Context Understanding. IEEE Trans. Intell. Transp. Syst..

[21] Bernhard W., Portmann P. Traffic simulation of roundabouts in Switzerland. Proceedings of the 2000 Winter Simulation Conference (Cat. No.00CH37165).

[22] Artesea G. Detecting and Tracking Vehicles in a Roundabout. https://pdfs.semanticscholar.org/a269/45eeeddaff654548520850d9dcdfe28ef7f4.pdf.

[23] Grejner-Brzezinska D.A., Toth C.K., Paska E., Tao C.V., Li J. (2007). Airborne remote sensing supporting traffic flow estimation. Advances in Mobile Mapping Technology.

[24] Zhao M., Kathner D., Jipp M., Soffker D., Lemmer K. Modeling driver behavior at roundabouts: Results from a field study. Proceedings of the 2017 IEEE Intelligent Vehicles Symposium (IV).

[25] Pedersini F., Sarti A., Tubaro S. (2001). Multi-camera parameter tracking. IEE Proc. Vision, Image Sign. Proces..

[26] Reulke R., Kroen A., Kahl T., Dalaff C., Schischmanow A., Schlotzhauer G. A Traffic Object Detection System for Road Traffic Measurement and Management. https://www.academia.edu/25487824/A_Traffic_Object_Detection_System_for_Road_Traffic_Measurement_and_Management.

[27] Zhu Z., Liang D., Zhang S., Huang X., Li B., Hu S. Traffic-Sign Detection and Classification in the Wild. Proceedings of the 2016 IEEE Conference on Computer Vision and Pattern Recognition (CVPR).

[28] Chen X., Ma H., Wan J., Li B., Xia T. Multi-view 3D Object Detection Network for Autonomous Driving. Proceedings of the 2017 IEEE Conference on Computer Vision and Pattern Recognition (CVPR).

[29] Yu Y., Li J., Wen C., Guan H., Luo H., Wang C. (2016). Bag-of-visual-phrases and hierarchical deep models for traffic sign detection and recognition in mobile laser scanning data. ISPRS J. Photogramm. Remote Sens..

[30] Geiger M., Lauer C., Wojek C., Stiller C., Urtasun R. (2014). 3D traffic scene understanding from movable platforms. IEEE Trans. Pattern Anal. Mach. Intell..

[31] Minaee S., Wang Y. Text extraction from texture images using masked signal decomposition. Proceedings of the 2017 IEEE Global Conference on Signal and Information Processing (GlobalSIP).

[32] Elqursh A., Elgammal A. (2012). Online Moving Camera Background Subtraction. Eur. Conf. Comput. Vision.

[33] Minaee S., Wang Y. (2019). An ADMM Approach to Masked Signal Decomposition Using Subspace Representation. IEEE Trans. Image Process..

[34] Hane C., Sattler T., Pollefeys M. Obstacle detection for self-driving cars using only monocular cameras and wheel odometry. Proceedings of the 2015 IEEE/RSJ International Conference on Intelligent Robots and Systems (IROS).

[35] Aoude G.S., Desaraju V.R., Stephens L.H., How J.P. Behaviour classification algorithms at intersections and validation using naturalistic data. Proceedings of the 2011 IEEE Intelligent Vehicles Symposium (IV).

[36] Clara Fang F., Castaneda H. (2018). Computer Simulation Modeling of Driver Behavior at Roundabouts. Int. J. Intell. Transp. Syst. Res..

[37] Deluka Tibljaš A., Giuffre T., Surdonja S., Trubia S. (2018). Introduction of Autonomous Vehicles: Roundabouts Design and Safety Performance Evaluation. Sustainability.

[38] Zhao M., Käthner D., Söffker D., Jipp M., Lemmer K. Modeling Driving Behavior at Roundabouts: Impact of Roundabout Layout and Surrounding Traffic on Driving Behavior. https://core.ac.uk/download/pdf/84275712.pdf.

[39] Código de Tráfico y Seguridad Vial. http://www.dgt.es/images/BOE-020_Codigo_de_Trafico_y_Seguridad_Vial.pdf.

[40] Molina M., Sanchez-Soriano J., Corcho O. (2015). Using Open Geographic Data to Generate Natural Language Descriptions for Hydrological Sensor Networks. Sensors.

[41] Redmon J., Divvala S.K., Girshick R.B., Farhadi A. You Only Look Once: Unified, Real-Time Object Detection. https://grail.cs.washington.edu/wp-content/uploads/2016/09/redmon2016yol.pdf.

[42] Cortes C., Vapnik V. (1995). Support-vector networks. Mach. Learn..

[43] Harrell F. (2001). Regression Modeling Strategies: With Applications to Linear Models, Logistic and Ordinal Regression, and Survival Analysis.

[44] Bengio Y., Courville A., Vincent P. (2013). Representation Learning: A Review and New Perspectives. IEEE Trans. Pattern Anal. Mach. Intell..

[45] Lightning Fast Data Science Platform for Teams. https://rapidminer.com/.

[46] Cherkassky V., Ma Y. (2004). Practical selection of SVM parameters and noise estimation for SVM regression. Neural Netw..

[47] Dosovitskiy A., Ros G., Codevilla F., López A., Koltun V. CARLA: An Open Urban Driving Simulator. http://vladlen.info/papers/carla.pdf.

